# HOME-BASED TRANSCRANIAL DIRECT-CURRENT STIMULATION AND EXPERIMENTAL PAIN SENSITIVITY

**DOI:** 10.1093/geroni/igz038.1227

**Published:** 2019-11-08

**Authors:** Hyochol Ahn, Chengxue Zhong, Setor Sorkpor, Hongyu Miao

**Affiliations:** University of Texas Health Science Center at Houston, Houston, Texas, United States

## Abstract

Osteoarthritis (OA) of the knee is one of the most common causes of pain in older adults. Clinic-based transcranial direct current stimulation (tDCS) is a noninvasive brain stimulation technique that has been shown to reduce pain, but no published studies have reported using home-based self-administered tDCS in older adults with knee OA. Thus, the purpose of this study was to examine the effect of home-based tDCS on experimental pain sensitivity in older adults with knee OA. Twenty community-dwelling participants aged 50–85 years with knee OA pain received ten daily sessions of 2 mA tDCS for 20 minutes at home. A multimodal quantitative sensory testing battery was completed, including heat pain tolerance, pressure pain threshold, and punctate mechanical pain. Participants (75% female) had a mean age of 61 years, and a mean body mass index in the sample was 28.33 kg/m2. All 20 participants completed all ten home-based tDCS sessions without serious adverse effects. The Wilcoxon Signed-Rank test showed that all the differences between the baseline measurements and experimental pain sensitivity measurements after 10 sessions were statistically significant. Effect sizes (Rosenthal’s R) were R = 0.35 for heat pain tolerance (P = 0.02), R = 0.40 for pressure pain threshold (P < 0.01), and R = 0.32 for punctate mechanical pain (P = 0.02). We demonstrated that home-based self-administered tDCS was feasible and reduced experimental pain sensitivity in older adults with knee OA. Future studies with well-designed randomized controlled trials are needed to validate our findings.

